# Heterogeneity in coronary heart disease risk

**DOI:** 10.1038/s41598-022-14013-3

**Published:** 2022-06-16

**Authors:** Cristoforo Simonetto, Susanne Rospleszcz, Jan Christian Kaiser, Kyoji Furukawa

**Affiliations:** 1 Institute of Radiation Medicine, Helmholtz Zentrum München German Research Center for Environmental Health (GmbH), Ingolstädter Landstraße 1, 85764 Neuherberg, Germany; 2 Institute of Epidemiology, Helmholtz Zentrum München German Research Center for Environmental Health (GmbH), Ingolstädter Landstraße 1, 85764 Neuherberg, Germany; 3 Institute for Medical Information Processing, Biometry and Epidemiology, Ludwig-Maximilians-Universität München, Marchioninistr. 15, 81377 Munich, Germany; 4 German Center for Cardiovascular Disease (DZHK), Partner Site Munich Heart Alliance, Munich, Germany; 5 Biostatistics Center, Kurume University, 67 Asahi-machi, Kurume, Fukuoka, 830-0011 Japan

**Keywords:** Cardiology, Diseases, Risk factors

## Abstract

There is large inter-individual heterogeneity in risk of coronary heart disease (CHD). Risk factors traditionally used in primary risk assessment only partially explain this heterogeneity. Residual, unobserved heterogeneity leads to age-related attenuation of hazard rates and underestimation of hazard ratios. Its magnitude is unknown. Therefore, we aimed to estimate a lower and an approximate upper bound. Heterogeneity was parametrized by a log-normal distribution with shape parameter σ. Analysis was based on published data. From concordance indices of studies including traditional risk factors and additional diagnostic imaging data, we calculated the part of heterogeneity explained by imaging data. For traditional risk assessment, this part typically remains unexplained, thus constituting a lower bound on unobserved heterogeneity. Next, the potential impact of heterogeneity on CHD hazard rates in several large countries was investigated. CHD rates increase with age but the increase attenuates with age. Presuming this attenuation to be largely caused by heterogeneity, an approximate upper bound on σ was derived. Taking together both bounds, unobserved heterogeneity in studies without imaging information can be described by a shape parameter in the range σ = 1–2. It substantially contributes to observed age-dependences of hazard ratios and may lead to underestimation of hazard ratios by a factor of about two. Therefore, analysis of studies for primary CHD risk assessment should account for unobserved heterogeneity.

## Introduction

**Figure 1:**
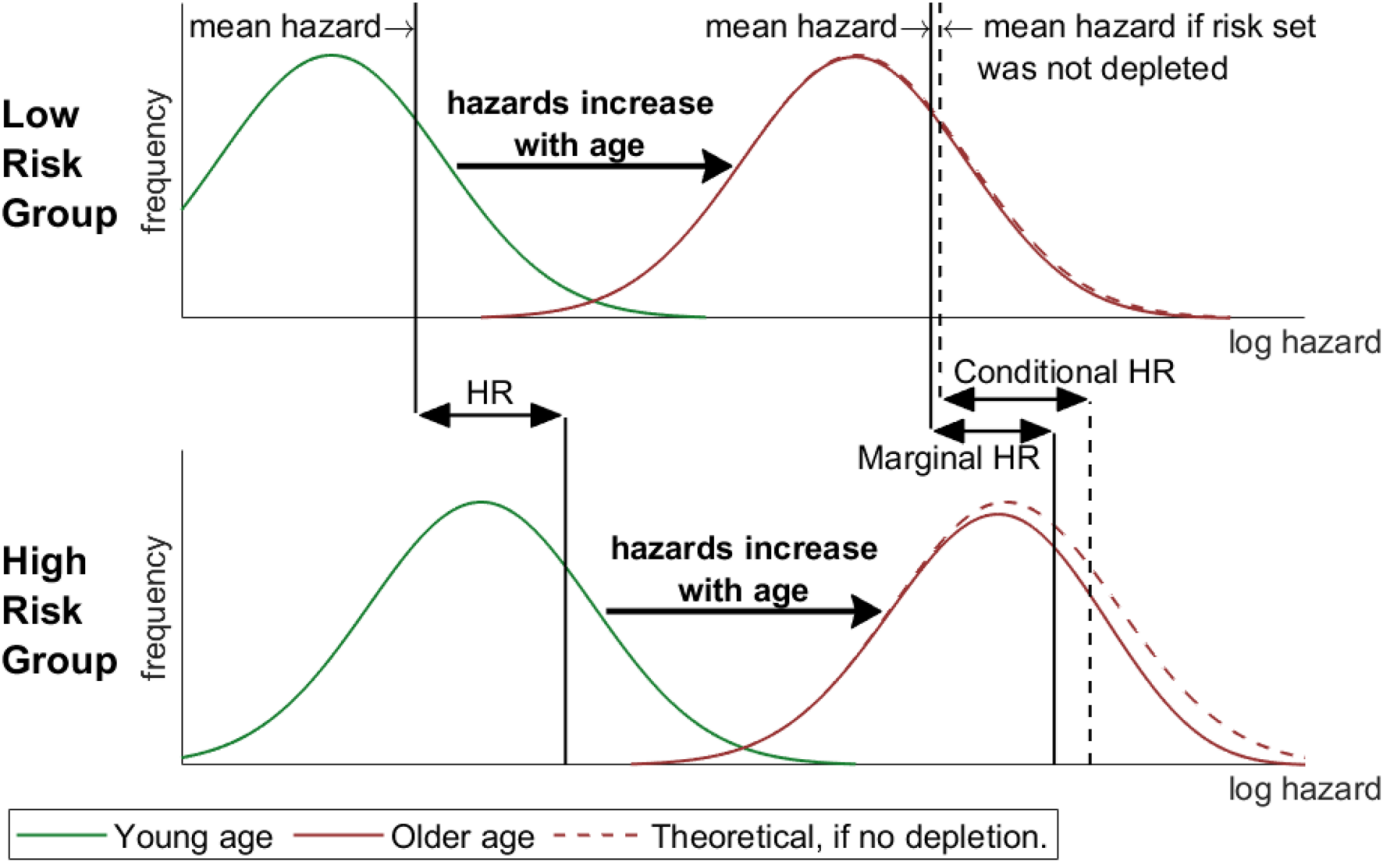
Unobserved heterogeneity attenuates hazard rates and ratios. The upper panel sketches the distribution of hazards within a low-risk group (e.g. non-smokers, normal blood pressure, normal cholesterol level,…). The green line refers to young, the red line to older age. The red dashed line shows the hypothetical distribution at older age if no CHD occurred: it is identical to the distribution at young age but shifted towards higher hazards. However, especially individuals with high hazards experience coronary heart disease and thus drop out of the risk set. The resulting depleted distribution is depicted as solid red line. Owing to the depletion, the mean hazard increases slower with age than individual risk. The bottom panel refers to a high risk group (e.g. smokers, normal blood pressure, normal cholesterol level,…). The marginal hazard ratio (HR) is the quotient of the mean hazards of two risk groups (e.g. smokers vs. non-smokers, each with normal blood pressure, normal cholesterol level,…). The conditional HR refers to the effect of a risk factor (e.g. smoking) on individual risk. At sufficiently low age, both HRs coincide. However, depletion of high-risk individuals is stronger in the high-risk group. This reduces the observed marginal HR with age.

Coronary heart disease (CHD) risk varies widely among individuals. Part of this variation can be explained by risk factors. However, true risk varies even among individuals with identical risk factor levels. This variation is called unobserved heterogeneity. Unobserved heterogeneity in risk estimation is inevitable, since no risk estimate can perfectly capture true CHD risk. However, from the statistical literature it is well known that unobserved heterogeneity can bias risk estimates and complicates the interpretation of hazards and hazard ratios (HRs)^1–3^. This problems have even led to the proposal to use other effect measures than HRs^4,5^. To understand the issue, a distinction has to be made between the so called marginal and the conditional hazard. Observed hazards always refer to risk groups specified by certain risk factor levels. Therefore, they are marginal with respect to unknown or unmeasured risk factors within the group. Instead, the conditional hazard can be thought of as an individual hazard: it cannot be determined as it depends on unmeasured factors. Assuming proportional hazards, observed marginal HRs are smaller than conditional HRs^5^. Therefore, marginal HRs underestimate the effect of risk factors in the individual. The underlying mechanism is depicted in Fig. 1: on average, individuals at higher risk for CHD experience an event earlier and thus drop out from the risk set. This reduces the mean risk within each risk group. Because this effect is stronger in high risk groups, also HRs are affected. Although this mechanism is more severe for common diseases^5^, it has not been thoroughly investigated so far in CHD epidemiology^6,7^. Aim of the present study is to estimate the size of unobserved heterogeneity and its consequences in CHD risk estimation. Analyses are based on published data from large studies and official WHO data.

As the name implies, there is no way to determine unobserved heterogeneity from incidence data in general. Some approaches employ familial risk or recurrent event data^2,8^ but are not generally applicable. Main goal of the present study is to establish a lower bound of unobserved heterogeneity in primary CHD risk assessment. The idea is obvious: The more risk factors are taken into account in a study, the larger part of heterogeneity can be captured. For an analysis that does not take the full set of risk factors into account, some of this heterogeneity remains unobserved. This part of unobserved heterogeneity can be estimated in the comprehensive analysis. Typically, this situation arises if assessment of some risk factors is expensive or limited by ethical concerns. In such case, the predictive value of risk factors may be known but they can still not be included in general risk prediction models.

Traditional CHD risk assessment is based on risk factors such as blood pressure, smoking behavior, and blood lipids^6,7^ which are subsumed to risk scores^9–11^. It has been difficult to increase the performance of traditional risk scores^12^, as most additional markers such as genetics or novel circulating biomarkers provide only modest improvements in prediction performance^13–16^. Improved prediction, however, can be obtained by diagnostic imaging data^17,18^. Coronary artery calcium (CAC) scanning has emerged as the most robust predictor of coronary events in the asymptomatic primary prevention population^19^ and even better discrimination can be obtained by coronary computed tomography angiography (CCTA)^20^. However, imaging is not routinely performed in primary risk assessment. Therefore, imaging in CHD risk assessment forms an example of the situation sketched above: heterogeneity assessable by CAC and CCTA remains unobserved and constitutes a lower bound of unobserved heterogeneity in traditional risk assessment.

Next, to provide a more complete picture, we also establish a rough upper bound of unobserved heterogeneity under some plausible presumptions. In population-based studies, it is typically observed that at young ages, risk strongly increases with age but the relative increase slows down at older age. Such attenuation of the hazard increase is predicted by unobserved heterogeneity. Based on published CHD mortality data, we will estimate the amount of heterogeneity that would be necessary to drive this attenuation. As several causes may contribute to the attenuation, the estimate constitutes an upper bound on unobserved heterogeneity.

Finally, the derived plausible range for heterogeneity is used to evaluate its impact on age- and sex-specific HRs. These results are compared to official WHO data. Implications of our findings on individual risk estimation are discussed.

## Methods

### Statistical methods

#### Parametrizing heterogeneity

CHD risk differs between individuals for different reasons. Differences are partially related to observed risk factors which we collectively call $R$. Therefore, $R$ may be thought of as a linear predictor involving several risk factors. Other, unobserved, factors $L$ are called latent. Making this distinction, we write the hazard $\lambda$ as

$$\lambda ={e}^{R+L}$$

This is called the *conditional* hazard. The term ${e}^{R}$ is the part that can be predicted for each individual in a study. On the other hand, ${e}^{L}$ is an individual factor (called frailty) that is not observed. The distribution of ${e}^{L}$ thus describes unobserved heterogeneity. We assume a log-normal frailty model^2^: $L$ is normally distributed (variance ${\sigma }_{L}^{2}$) and independent of $R$ and normalized at young age by $E[{e}^{L}]=1$. For simplicity, we will assume also $R$ to be normally distributed (variance ${\sigma }_{R}^{2}$). Then, variances simply add to yield the total variance of the log hazards ${\sigma }^{2}={\sigma }_{R}^{2}+{\sigma }_{L}^{2}$ .

While the conditional hazard describes individual risk, the *marginal* hazard $\overline{\lambda }$ describes the average hazard in some group or population, thus averaging over $L$:

$$\overline{\lambda }={e}^{R} E[{e}^{L}]$$

As illustrated in Fig. 1, the marginal hazard deviates from the conditional hazard with increasing age because individuals with high ${e}^{L}$ are depleted from the risk set. Stronger attenuation occurs for larger cumulative hazards thus affecting HRs. The mathematical derivation is presented in the Appendix.

#### Estimating explained heterogeneity from the concordance index

Without access to the original individual data, the variance ${\sigma }_{R}^{2}$ must be estimated from published summary data. Good prediction performance means large explained heterogeneity. Therefore, ${\sigma }_{R}^{2}$ is related to the area under the receiver-operator curve (AUC) and for normally distributed explanatory variables in logistic regression, the following formula approximately holds^21^

1 $$AUC=\Phi ({\sigma }_{R}/\sqrt{2})$$

with $\Phi$ denoting the cumulative normal distribution. We use this formula to calculate ${\sigma }_{R}^{2}$ from published AUC or concordance index values.

In particular, we are interested in studies investigating the incremental prognostic value of some imaging marker. Equation (1) is thus applied twice: First, for the risk prediction function without the imaging marker, and second, for the function with the marker added. This results in two estimates of ${\sigma }_{R}^{2}$. For the risk prediction function that takes into account imaging, a larger part of heterogeneity can be explained, related to larger ${\sigma }_{R}^{2}$. The difference of the two estimates corresponds to the additional heterogeneity which is assessable by diagnostic imaging data.

### Data

#### Using imaging studies to establish a lower bound of heterogeneity

To estimate the part of CHD risk heterogeneity that can be assessed by imaging but not by traditional risk factors, relevant studies from the literature were collated. Following the available evidence, first the improvement of risk estimation due to CAC scoring was investigated, followed by the additional improvement by use of CCTA.

For CAC, long-term population based studies have shown the incremental prognostic value of CAC scoring. For the two largest studies^22^, MESA and HNR, concordance index values were jointly published in^23^ together with results of the DHS. The discriminative ability of CAC was superior compared to other non-traditional risk markers^24,25^. We therefore use the studies published in^23^ for analysis, as well as a recent clinical study because of its large study size^26^.

For CCTA, a PubMed search was performed for CHD studies assessing the concordance index (or AUC) from CCTA, using reference tracking and the “similar articles” feature of PubMed. As only a single population based study could be identified^27^, we list also clinical studies. However, the treating physicians were not blinded for imaging results. This may have increased the rate of interventions in patients with adverse findings on CCTA. On the one hand, this may have led to an apparent improvement in discrimination for cardiac interventions. On the other hand, it may have reduced the rate of cardiac deaths and myocardial infarctions thus leading to an apparent decline in discrimination for hard endpoints.

As derivation of a precise estimate is therefore difficult, we place more emphasis on population based studies and rather use a conservative estimate as given by the lower values from the calculations in Eq. (1). Combining results from CAC and CCTA, we then derive heterogeneity assessed only by imaging. This constitutes a lower bound for unobserved heterogeneity for traditional primary risk assessment.

#### Using WHO mortality rates to establish a rough upper bound of heterogeneity

We analyze sex-specific crude CHD mortality rates for four different large countries (USA, Russian Federation, Japan, Germany) as published in the WHO Mortality Data Base^28^. Countries were chosen for existence of complete and continuous data, aiming for a wide range of CHD rates and main risk factors, and aiming for some overlap with the studies on CAC and CCTA. In order to gauge period and cohort effects, rates are presented for the first and last year for which CHD rates were available defined by ICD-9 or ICD-10. Based on the geometric mean of data from this first and last year, we extrapolate exponentially the trend from younger ages (30–45 years). This extrapolation constitutes a hazard age dependence without any attenuation. Assuming this age dependence to hold for the conditional hazard, we calculate the attenuation induced by unobserved heterogeneity for different values of the shape parameter σ, see Eq. (A.2). The resulting, attenuated hazard curve is visually compared to the empirical data in the older age groups. This way, a value for σ can be roughly estimated for which the observed attenuation can be explained from unobserved heterogeneity. However, as attenuation might also be due to other causes, this estimation gives an upper bound. Because the analysis is based on crude rates, unobserved heterogeneity coincides with total heterogeneity (${\sigma }_{L}=\sigma$). In general, ${\sigma }_{L}\le \sigma$, and the upper bound thus holds also for studies including traditional risk factors.

#### Statement

All methods were carried out in accordance with relevant guidelines and regulations.

## Results

### CHD risk heterogeneity captured by imaging

Characteristics of studies analyzing the incremental value of CAC scoring on traditional risk prediction are listed in Table 1. For each study two estimates of the observed variance ${\sigma }_{R}^{2}$ are presented as derived from the concordance indices. Variance ${\sigma }_{T}^{2}$ relates to a risk estimation based on traditional risk factors only, and ${\sigma }_{T}^{2}+{\sigma }_{CAC}^{2}$ to the estimation also incorporating CAC. These estimates always refer to the endpoint of the respective study. As can be seen from Table 1, ${\sigma }_{T}^{2}$ varied between studies from 0.7 to 1.3. CAC scoring increased the variance of predicted risk by 0.38 to 0.51.

**Table 1:** Studies investigating the incremental prognostic value of coronary artery calcium (CAC) scoring.

Study	Cohort recruitment	Endpoint	Cases/participants; follow up	${\sigma }_{T}^{2}$	${\sigma }_{CAC}^{2}$
McClelland^23^ (Multi-Ethnic Study of Atherosclerosis)	Population based, age 45–84, USA	CHD death, myocardial infarction, resuscitated cardiac arrest, revascularization after angina	422/6726; 10.2y (median)	0.91	0.51
McClelland^23^ (Heinz Nixdorf Recall Study)	Population based, age 45–75, Germany		274/3692; 10.4y (median)	0.68	0.50
McClelland^23^ (Dallas Heart Study)	Population based, age 45–65, Texas		58/1080; 9.3y (median)	1.21	0.41
Blaha^26^ (Coronary Artery Calcium Consortium)	Asymptomatic individuals referred to clinical CAC scoring, age 45–79, USA	CHD death	421/53,487; 12y (mean)	1.30	0.38

Studies and estimated variances of the log risks predicted by traditional risk factors (${\upsigma }_{\mathrm{T}}^{2}$) and additional variance by CAC scoring (${\upsigma }_{\mathrm{CAC}}^{2}$). For all studies, traditional risk factors include age, sex, smoking, systolic blood pressure, anti-hypertensive medication, total cholesterol, high-density lipoprotein cholesterol, lipid-lowering medication, diabetes, family history, and ethnicity.

*CHD* Coronary heart disease.

Table 2 shows the studies which investigate the incremental prognostic value of CCTA as compared to risk assessment by traditional risk factors and CAC scoring. Limitations of these studies have already been detailed above, and may explain divergent variances comparing for example the two studies of Hadamitzky et al.^29,30^, which are based partially on the same patient cohort. However, an estimate ${\sigma }_{CCTA}^{2} \sim 0.5$ appears to be rather conservative, in particular as the studies listed in Table 2 may not have assessed all relevant features assessable by modern CCTA^20,31^.

**Table 2:** Studies investigating the incremental prognostic value of coronary computed tomography angiography (CCTA).

Study	Cohort recruitment	Endpoint	Cases/participants; follow up	Features assessed by CCTA	${\sigma }_{T+CAC}^{2}$	${\sigma }_{CCTA}^{2}$
Moon^27^	Population based, age 65+, South Korea	Cardiac death, MI	24/470; 8.2y (median)	Stenosis (modified Duke score^32^)	0.81	0.56
Halon^33^	Type 2 diabetics, age 55–74, Israel	Cardiovascular death, MI, unstable or new-onset angina requiring intervention	41/630; 6.6y (mean)	Plaques (relative volume), stenosis (Gensini score^34^)	1.03	0.71
Hadamitzky^29^	Suspected CHD, Germany	Cardiac death, MI, unstable angina requiring hospitalization, late coronary revascularization	47/2223; 2.4y (median)	Stenosis severity	1.98	1.03
Hadamitzky^30^	Suspected CHD, Germany	Cardiac death, MI	25/1584; 5.5y (median)	Number of segments with stenosis ≥ 25% or any plaques	0.63	0.24
Hou^35^	Suspected CHD, China	Cardiac death, MI, late coronary revascularization	363/4425; 3.0y (median)	Number of obstructive vessels, occlusion, plaque composition, location	1.68	2.7
Nadjiri^36^	Suspected CHD	Cardiac death, MI, late coronary revascularization	46/1168; 5.7y (median)	Segment stenosis score^37^, low attenuation plaque volume	1.28	0.56

Estimated variances of the log risks refer to risk predicted by traditional risk factors and coronary artery calcium (CAC) scoring (${\upsigma }_{\mathrm{T}+\mathrm{CAC}}^{2}$) and to the additional variance obtained by CCTA imaging (${\upsigma }_{\mathrm{CCTA}}^{2}$).

*CHD* coronary heart disease, *MI* myocardial infarction.

In summary, the variance of the log hazards assessable by imaging, ${\sigma }_{CAC}^{2}+{\sigma }_{CCTA}^{2}$, is of the order of 1 or larger. Without imaging information, this part of the variance would not have been explained and thus contributed to unobserved heterogeneity. For studies based on traditional risk factors only, unobserved heterogeneity may thus be described by a variance of log hazards ${\sigma }_{L}^{2}\ge$ 1, or equivalently by a shape parameter ${\sigma }_{L}\ge$ 1.

### CHD risk heterogeneity and attenuation of hazard rates

**Figure 2:**
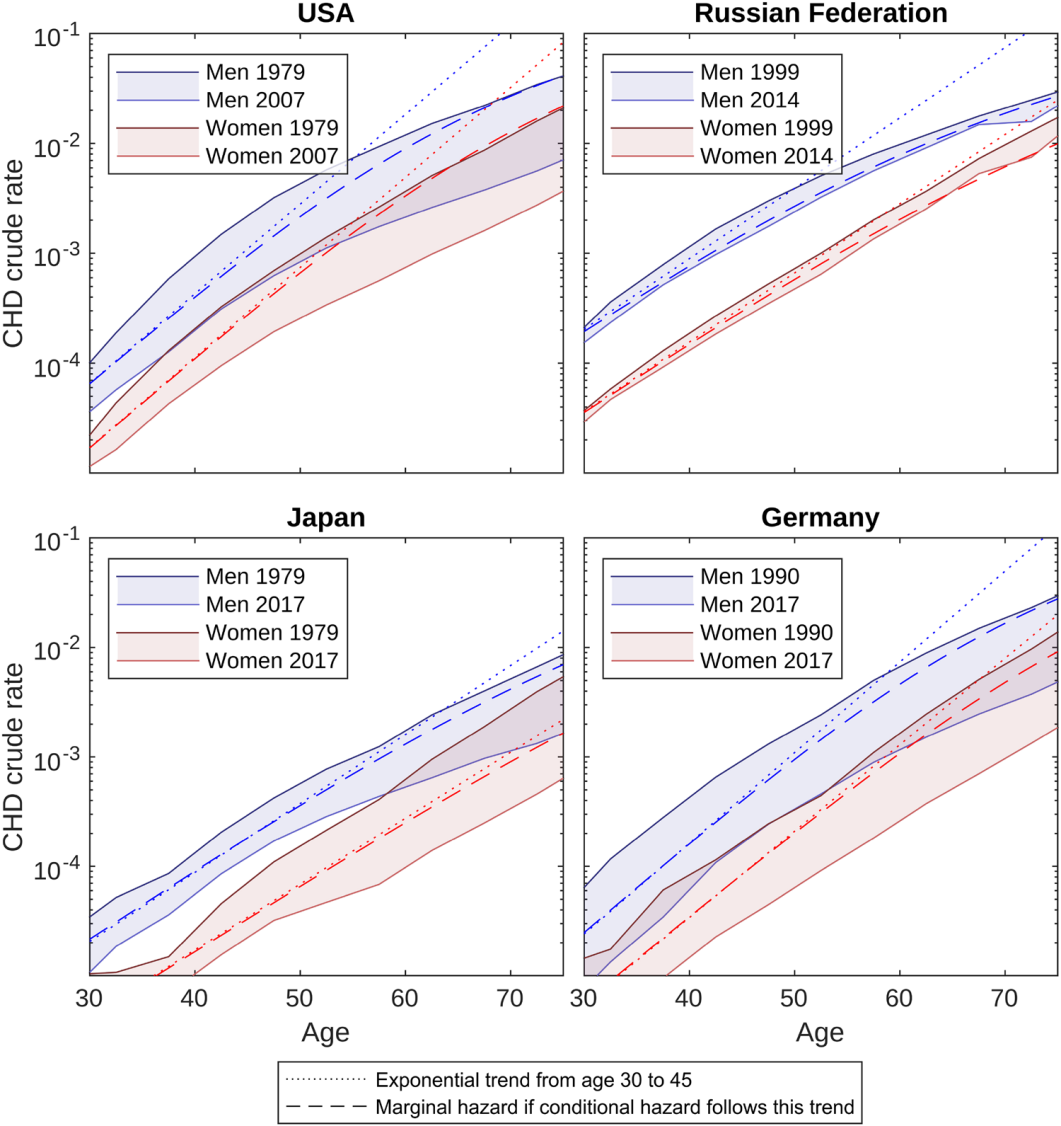
Coronary heart disease (CHD) crude mortality rates according to the WHO Mortality Data Base. To guide the eyes, the area between mortality rates of different calendar years has been shaded. Dashed lines illustrate marginal hazards resulting from unobserved heterogeneity with shape parameter $\upsigma =2$ assuming exponentially increasing conditional hazards as delineated with dotted lines.

Based on the WHO Mortality Data Base^28^, Fig. 2 shows crude CHD mortality rates for four large countries. Dotted lines show the exponentially extrapolated trend from ages 30 to 45. The deviation between the true CHD rates and the extrapolated trend illustrates the attenuation of hazard rates with age. Obviously, attenuation affects different rates to different degrees: For women in Japan, the extrapolation reasonably describes also rates for older ages. For men and for other countries, there is more attenuation from the extrapolated trend with higher ages, and generally attenuation is stronger for men than women. Stronger attenuation for larger cumulative hazards exactly corresponds to the behavior predicted by heterogeneity.

To estimate the heterogeneity required to drive this attenuation, we calculated the marginal hazards for conditional hazards that follow the exponential trend. In other words, no attenuation was assumed at the individual level. The dashed lines show the marginal hazards presuming a shape parameter $\sigma =2$. As heterogeneity has minor impact for small cumulative hazards, marginal and conditional hazard are similar for women in Japan. For women in the Russian Federation, the marginal hazard attenuates even stronger than the crude rates. Overall, heterogeneity with $\sigma =2$ largely, but not fully suffices to explain the attenuation.

If presuming $\sigma =3$ in the above calculation instead of $\sigma =2$, heterogeneity alone sufficed to explain the observed attenuation for USA and Germany, and led to even stronger than observed attenuation for the Russian Federation and Japan (not shown).

In summary, heterogeneity can explain why higher hazard rates are associated with stronger attenuation. For this explanation a shape parameter $\sigma \sim 2$ is about sufficient. However, as we do not expect heterogeneity to be the only explanation for attenuation, $\sigma \sim 2$ presents an upper bound for total heterogeneity.

Taken together, in “CHD risk heterogeneity captured by imaging” a lower bound of ${\sigma }_{L}=$ 1, was established for unobserved heterogeneity for studies lacking imaging information. A rough upper bound $\sigma \sim$ 2 was estimated for total heterogeneity in the previous section. Since heterogeneity explained by traditional risk factors can be approximately described by ${\sigma }_{R} \sim$ 1 (see Table 1), and shape parameters add quadratically, ${\sigma }^{2}={\sigma }_{R}^{2}+{\sigma }_{L}^{2}$, this implies a plausible range ${\sigma }_{L}=$ 1–2 for unobserved heterogeneity in studies based on traditional risk factors only.

## Discussion

Unobserved heterogeneity in the range ${\sigma }_{L}=$ 1–2 has substantial impact on HRs and thus on individual risk estimation. We will now illustrate by specific worked examples how risk is underestimated if heterogeneity is not taken into account. To this aim, we use data from the WHO CVD Risk Chart Working Group^38^.

Recall that in the presence of unobserved heterogeneity, it is important to distinguish the conditional from the marginal HR. The conditional HR cannot be observed directly and relates to comparing two individuals who differ *only* in some risk factor(s) under investigation. The marginal HR can be observed as it relates to comparing two groups which differ in the investigated risk factor(s) but may also differ in other, unknown or unmeasured covariates. The prevalence of these other covariates is age dependent, thus reducing the marginal HR with age, as illustrated in Fig. 1.

**Figure 3:**
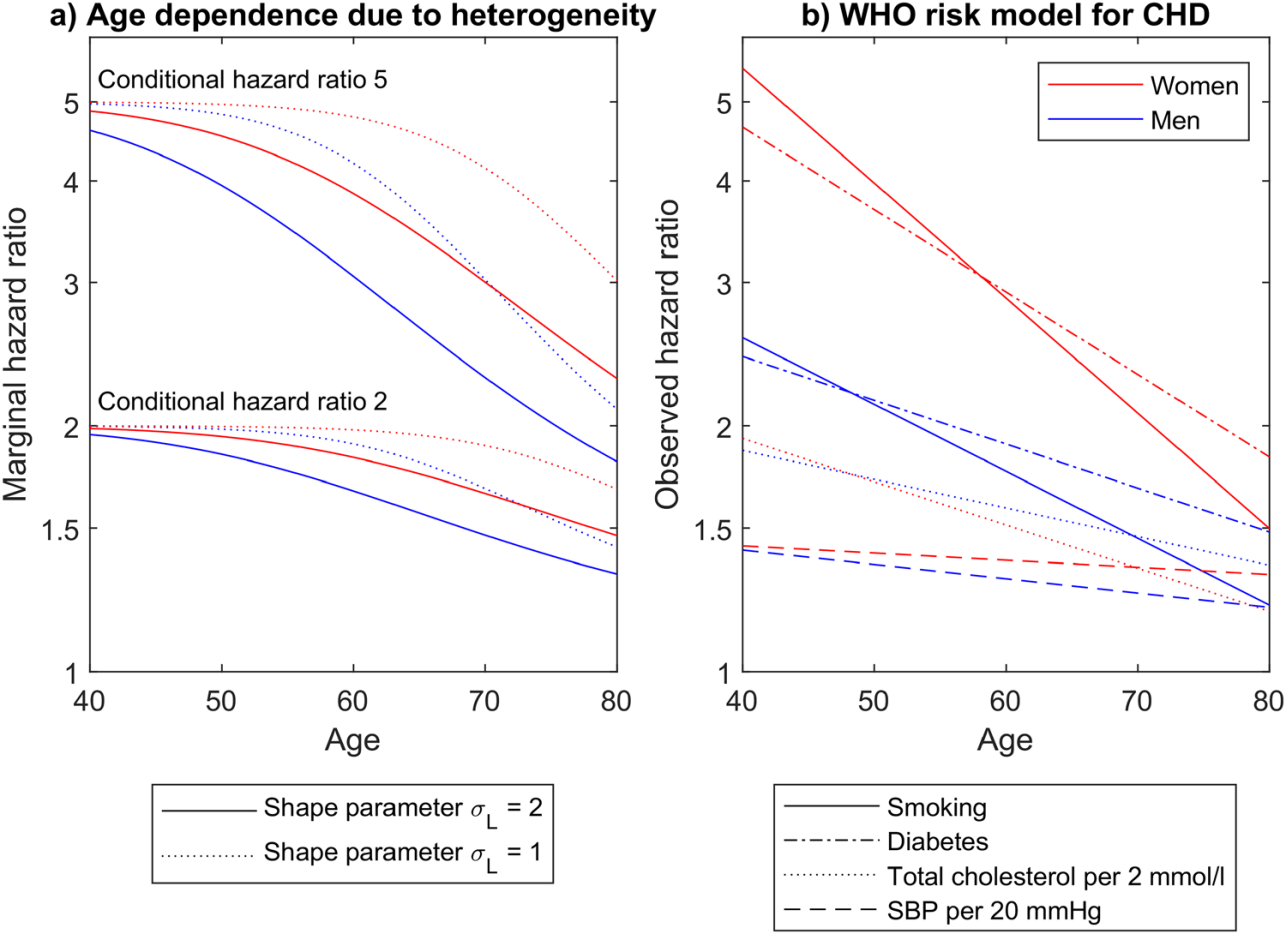
Predicted and observed age dependence of hazard ratios. (**a)** Heterogeneity induced age dependence of the marginal hazard ratio for constant conditional hazard ratio values of 5 (lines originating at the top left) and 2 (other lines). Two different values of the shape parameter ${\upsigma }_{\mathrm{L}}$ were applied for unobserved heterogeneity. Exponentially increasing conditional hazards were assumed as shown in Fig. 2 for Germany. (**b)** Age dependence of the hazard ratios of several risk factors for 10-year cardiovascular risk, as derived by the WHO CVD Risk Chart Working Group^38^. In both panels, red lines refer to women, blue lines to men.

Figure 3 now juxtaposes the age dependence of HRs expected from unobserved heterogeneity with empirical data. The left panel shows the age dependence of marginal HRs due to unobserved heterogeneity, i.e. assuming constant conditional HRs. To calculate the effect of depletion of high risk individuals, Eq. (A.3), sex-specific exponentially increasing German CHD mortality rates (see Fig. 2) were applied. As can be seen, attenuation of the marginal HRs is stronger for stronger heterogeneity (${\sigma }_{L}= 2$ vs. ${\sigma }_{L}= 1$), for larger conditional HRs (5 vs. 2), and for larger cumulative hazards (men vs. women).

The right panel relates to results from the WHO CVD Risk Chart Working Group and is based on CHD mortality and myocardial infarction in 376.177 individuals from 85 different cohorts^38^. It shows sex-specific HRs for smoking, diabetes, cholesterol, and blood pressure. Obviously, all HRs decrease with age, and larger HRs tend to attenuate stronger. For diabetes and smoking, HRs were larger for women than for men. Overall, comparing the two figure panels, it can be concluded that heterogeneity may explain substantial part of the observed age dependence, especially for ${\sigma }_{L}= 2$.

However, this has profound impact on the interpretation of the HRs. For a risk factor that increases individual risk by a factor 5 (conditional HR), only a HR of about 2.5 may be observed at age 65 (marginal HR), see Fig. 3a. Vice versa, the observed age-related decrease of HRs (see Fig. 3b) may be dominated by depletion of high-risk individuals; there may be no significant reduction in individual risk with age. As a main goal of accurate CHD risk estimation is to improve patient communication to motivate healthy lifestyle choices, the conditional HR is the more relevant measure in this context. As exemplified above, it may be twice as large as the marginal HR.

The marginal HR is the relevant measure e.g. to determine the number of deaths attributable to a risk factor. It can be derived directly from incidence data. The above worked example also has implications with regard to the marginal HR: As illustrated in Fig. 3a, a sigmoidal age dependence is expected. The exponential dependence assumed in the WHO model therefore overestimates HRs at young ages.

Estimates of heterogeneity explained by CAC and CCTA varied between studies. Partially, this may be explained by imaging results to have influenced treatment decisions and therefore risk. Moreover, studies differed in the features derived from CCTA, likely leading to different prognostic values. Therefore, rather conservative estimates were applied. Results in this study were based on some simplifying assumptions. The choice for log-normal distributions was motivated by the widespread use of the log-transformation to relate the hazard with a linear predictor. The distribution of some particular risk factor may be far from normal. However, there are many relevant risk factors in CHD such that the linear predictor may be expected to be distributed approximately normally. Moreover, the distribution of the log CAC scores indeed appears to be approximately normally distributed^39^. In our analysis we have presumed a constant frailty. However, it may vary with age due to varying biological or environmental factors, possibly related to prevention measures. Also randomness may play a role in individual disease development^1^. In this regard, it should be noted that imaging data can predict risks for a decade as can be seen from Table 1, thus indicating limited relevance of variation with time. Moreover, in agreement to the present study, large heterogeneity was obtained in a recent modeling study that was based on autopsy data in youth and incorporated randomness^40^. Finally, it should be noted that even if individual frailties are constant, the variance of unobserved heterogeneity is not. The depletion of high-risk individuals gradually reduces total variance and thus also unobserved heterogeneity^2^. For example, assume the conditional hazard for German men, Fig. 2, and presume a log-normal frailty model at birth with shape parameter ${\sigma }_{L}= 2$. Then at higher age, heterogeneity is not strictly log-normal any more, and at age 70 the standard deviation of the log hazards is not 2 but only 1.8. In any case, risk estimates are mostly relevant for middle and old age for which our estimates were derived.

Our analysis showed that the attenuation of the increase of CHD hazard rates can be explained by unobserved heterogeneity. Based on this conclusion we have argued that $\sigma$ may not be too large in order not to yield a too strong attenuation. However, this argument has a loophole. Even though it may appear contrived, it is not excluded mathematically that the conditional hazard may grow even faster than exponentially. For this case, our upper bound would be evaded. Therefore, we performed no stringent evaluation to derive the upper bound but estimated it by visual inspection. Also no competing causes of death were considered in the present study. For given frailty and shared risk factors, competing causes of death would reinforce the attenuations.

With access to primary data, some of the presented results could have been obtained directly. This includes the distributions of predicted hazards, which were here assumed to follow log-normal distributions. Primary epidemiological data can easily be analyzed with frailty models^2^. As shown here, this would be important for interpretation of hazard ratios and for extrapolation to young ages. To evaluate the potential impact of unobserved heterogeneity, we suggest log-normal frailty models with ${\sigma }_{L}$ in the range 1–2. When establishing new risk models, sub-studies including additional risk factors may help to derive more specific lower bounds on unobserved heterogeneity.

## Conclusions

Additional markers, such as imaging, improve CHD risk prediction beyond traditional risk factors. This implies the existence of unobserved heterogeneity in studies based on traditional risk factors only. Unobserved heterogeneity can attenuate hazard ratios towards 1, and according to our estimates this attenuation is substantial. Observed hazard ratios may underestimate actual individual risk by a factor of two. Therefore, even if risk scores reliably predict absolute risks, they may fail to predict the impact of a risk factor on individual risk. However, the impact on individual risk is important for risk communication and to motivate healthy lifestyle changes in primary prevention. Therefore, frailty models should be applied in studies used for primary risk assessment.

## Supplementary Information

Supplementary Information.

## Data Availability

All data generated or analysed during this study are included in this published article.

## Abbreviations

AUC: Area under the receiver-operator curve
CAC: Coronary artery calcium
CCTA: Coronary computed tomography angiography
CHD: Coronary heart disease
HR: Hazard ratio
