# Supplementary material for: Heterogeneity in coronary heart disease risk
Source: Sci Rep. 2022 Jun 16;12:10131. doi: 10.1038/s41598-022-14013-3 (PMC9203574; doi:10.1038/s41598-022-14013-3)
Supplement: Supplementary file 1 — Supplementary Information. [file 41598_2022_14013_MOESM1_ESM.pdf]

Appendix to:
Heterogeneity in Coronary Heart Disease Risk

Cristoforo Simonetto, Susanne Rospleszcz, Jan Christian Kaiser, Kyoji Furukawa

The existence of latent factors L has impact on the age dependence of observed survival and hazard functions (1). To study this impact, we explicitly write the dependence of the conditional hazard λ on age a and observed risk factors r:

$$\lambda(a, r|L) = \lambda(a, r)\, e^L$$

The corresponding conditional survival is given by

$$S(a, r|L) = \exp\left(-e^L \int_0^a \lambda(t, r)\, dt\right) \qquad \text{(Eq. A.1)}$$

Survival is reduced for large cumulative $\lambda(a, r)$ and for large frailty e^L. For given age and observed risk factors, individual differences in survival are stronger for broad distribution of the frailty e^L. The depletion of high-risk individuals is reflected in the marginal hazard by inclusion of the conditional survival in the expectation value:

$$\bar{\lambda}(a, r) = \frac{E[\,\lambda(a,r|L)\, S(a,r|\, L)]}{E[S(a,r|\, L)]} = \lambda(a, r)\frac{E[\exp(L)\, S(a,r|\, L)]}{E[S(a,r|\, L)]} \qquad \text{(Eq. A.2)}$$

As the contribution of individuals with high frailty e^L diminishes, the marginal hazard attenuates with age, i.e. $\bar{\lambda}(a, r)/\lambda(a, r)$ decreases with age. The attenuation is stronger for larger λ, see Eq. A.1. Therefore, also HRs comparing different levels of risk factors r_1, r_2, attenuate:

$$\frac{\bar{\lambda}(a, r_1)}{\bar{\lambda}(a, r_2)} = \frac{\lambda(a, r_1)}{\lambda(a, r_2)}\frac{E[\exp(L)\, S(a, r_1|\, L)]}{E[\exp(L)\, S(a, r_2|\, L)]}\frac{E[S(a, r_2|\, L)]}{E[S(a, r_1|\, L)]} \qquad \text{(Eq. A.3)}$$

This means for $\frac{\bar{\lambda}(a, r_1)}{\bar{\lambda}(a, r_2)} > 1$, this fraction to become smaller than $\frac{\lambda(a, r_1)}{\lambda(a, r_2)}$ with advancing age. The conditional HR $\lambda(a, r_1)/\lambda(a, r_2)$ represents the HR for two individuals that differ only in the level $r_1$vs. r_2 of some risk factor(s). In contrast, the marginal HR applies to groups with levels r_1 vs. r_2. Because hazards cannot be determined for individuals, only the marginal hazard and the marginal HR can directly be deduced from incidence data.

References

1. Balan TA, Putter H. A tutorial on frailty models. Stat Methods Med Res. 2020;29(11):3424-54.
